# Visualizing the 3D Architecture of Multiple Erythrocytes Infected with *Plasmodium* at Nanoscale by Focused Ion Beam-Scanning Electron Microscopy

**DOI:** 10.1371/journal.pone.0033445

**Published:** 2012-03-14

**Authors:** Lia Carolina Soares Medeiros, Wanderley De Souza, Chengge Jiao, Hector Barrabin, Kildare Miranda

**Affiliations:** 1 Laboratório de Ultraestrutura Celular Hertha Meyer, Instituto de Biofísica Carlos Chagas Filho, Universidade Federal do Rio de Janeiro, Rio de Janeiro, Rio de Janeiro, Brazil; 2 Instituto Nacional de Ciência e Tecnologia em Biologia Estrutural e Bioimagens, Universidade Federal do Rio de Janeiro, Rio de Janeiro, Rio de Janeiro, Brazil; 3 Laboratório de Membranas Transportadoras, Instituto de Bioquímica Médica, Universidade Federal do Rio de Janeiro, Rio de Janeiro, Rio de Janeiro, Brazil; 4 Laboratório de Biotecnologia, Diretoria de Programas, Instituto Nacional de Metrologia, Normalização e Qualidade Industrial (INMETRO), Xerém, Rio de Janeiro, Brazil; 5 FEI Company, Eindhoven, The Netherlands; Bernhard Nocht Institute for Tropical Medicine, Germany

## Abstract

Different methods for three-dimensional visualization of biological structures have been developed and extensively applied by different research groups. In the field of electron microscopy, a new technique that has emerged is the use of a focused ion beam and scanning electron microscopy for 3D reconstruction at nanoscale resolution. The higher extent of volume that can be reconstructed with this instrument represent one of the main benefits of this technique, which can provide statistically relevant 3D morphometrical data. As the life cycle of *Plasmodium* species is a process that involves several structurally complex developmental stages that are responsible for a series of modifications in the erythrocyte surface and cytoplasm, a high number of features within the parasites and the host cells has to be sampled for the correct interpretation of their 3D organization. Here, we used FIB-SEM to visualize the 3D architecture of multiple erythrocytes infected with *Plasmodium chabaudi* and analyzed their morphometrical parameters in a 3D space. We analyzed and quantified alterations on the host cells, such as the variety of shapes and sizes of their membrane profiles and parasite internal structures such as a polymorphic organization of hemoglobin-filled tubules. The results show the complex 3D organization of *Plasmodium* and infected erythrocyte, and demonstrate the contribution of FIB-SEM for the obtainment of statistical data for an accurate interpretation of complex biological structures.

## Introduction

Malaria is one of the most deadly infectious diseases worldwide, responsible for 247 million cases and 881 thousand deaths (85% of deaths occurring in children under 5 years old) annually in more than 100 countries. Despite the efforts of World Health Organization, half of the world population is still at risk of malaria infection [1]. *Plasmodium*, the causal agent of malaria, is an obligatory intracellular parasite that grows and divides inside a parasitophorous vacuole in the red blood cell [2]. Because the mature erythrocyte has no intracellular organelles and none of the trafficking machinery used by most eukaryotic cells to transfer proteins to their correct destinations, the parasite needs to set up a completely novel system for trafficking of proteins and antigens to the surface of red blood cells [3], [4]. This system includes insertion of membrane profiles in the cytoplasm of the erythrocyte, such as membrane clefts, named Maurer's clefts in *P. falciparum* [5] or membrane clefts in other species such as the human parasite *P. vivax* [6] and rodent malaria parasite *P. berghei* [7], and a tubovesicular network, a term that has been introduced for a membranous network formed by projections of the parasitophorous vacuole membrane (PVM), described in *P. falciparum* [8], *P. vivax* [6] and *P. berghei* [7]. An association between these membranous structures with alterations on the surface of infected erythrocyte has been demonstrated by Bhattacharjee et al [9].

Although the molecular biology of malaria parasites is well characterized, the knowledge concerning the ultrastructural features of the *Plasmodium* and the infected red blood cells remains sketchy. Different microscopy techniques have been used to achieve 3D visualization of intraerythrocytic forms of *Plasmodium*, such as the 3D reconstruction of serial sections of the mitochondrion of *P. falciparum* [10], the feeding apparatus of *P. falciparum*, *P. berghei* [11], [12] and *P. chabaudi* [13], the apicoplast of *P. falciparum* [14] and the membrane features in the cytoplasm of the erythrocytes infected with *P. falciparum* [15].

Micro and nanoscale three dimensional visualization has been a challenge in life sciences. Multiple strategies to reconstruct cell volumes have been applied and, among the techniques currently available, electron microscopy has played a key role. Electron tomography and 3D reconstruction from serial sections comprise the two main methods that have been applied to reconstruct and visualize different biological structures with nanoscale resolution. Both techniques, however, present a number of limitations when high resolution information on the 3D organization of large volumes is required. In 3D reconstruction from serial sections [16]–[18], a ribbon of consecutive sections is obtained and profile images of the same object in different sections are recorded for subsequent alignment and reconstruction. Due to the limited number of consecutive sections that can be collected from the EM grid, only volumes up to a maximum of a few microns can be reconstructed. In addition, as the 2D images obtained are formed by a projection of the section volume, the zeta (z) resolution is limited by the section thickness (that usually varies from 50 to 200 nm). In electron tomography, on the other hand, the reconstructed volumes are calculated from an aligned tilt series that contains a sequence of projected images of the same object, recorded from different angles. This provides higher x-y and z resolution and high quality 3D reconstructions. However, as the object is usually embedded in a section or prepared as a whole mount (in the case of macromolecular assemblies or thin cells), the extent of the volume that can be reconstructed is limited by the section thickness (up to 500 nm, in conventional ET, in a 300 kV microscope) or by the specimen thickness itself. Alternative strategies such as the combination of serial sectioning and ET [19]–[22] or the acquisition of tomograms using scanning transmission electron microscopy [23], [24], have increased the sample volume that can be reconstructed, without a significant loss in resolution, to a few microns in the first case and up to 1 µm in the later.

FIB-SEM microscopy has been shown to be a powerful tool for the study of various types of materials, such as metal, ceramics, and more recently, biological materials [25]–[28]. FIB-SEM is a combination of a scanning electron microscope (SEM) and a focused ion beam (FIB) that coincides at its focal point. This combination enables bulk samples to be locally modified by ion milling, exposing the deeper regions of the material, which are subsequently imaged at high resolution with the electron beam. This has been used to expose internal surfaces of a variety of biological models such as arthropods [29], teeth [30]–[32], as well as soft and organic samples [33], including arteries and atherosclerotic tissues [34].

The ability to carry out high resolution milling and subsequent imaging of different sample materials has transformed the FIB-SEM into a promising tool for 3D reconstruction, especially if the microscope is equipped with a high performance back-scattered electron detection system, where metal stained/labeled structures such as osmium-stained membranes can be easily visualized [35]–[37]. In this case, a sequence of alternating sectioning with the ion beam and imaging with the electron beam generate the 3D data. As this process is done within the microscope chamber, the extent of the volume that can be reconstructed is much larger than with the TEM reconstruction methods and, given the proper orientation of the sample, theoretically dependent of the sample stability and time dedicated to milling and image acquisition within the microscope chamber. As a consequence, a large number of cells and intracellular structures can be modeled in 3D and combined surface and intracellular information can be obtained. Since 3D information of a large number of cells and intracellular structures is obtained, analyses of different parameters related to the structural organization (average number, volume, surface to volume ratio, e.g.) of cells and organelles may retrieve statistically relevant data.

In this work, we report the use of FIB milling followed by low voltage backscattered imaging to carry out 3D reconstruction of multiple erythrocytes infected with *Plasmodium chabaudi*. Serial images obtained from the surface of an epoxide block containing glutaraldehyde-osmium fixed epon-embedded samples, followed by 3D modeling showed a series of modifications on the surface of the erythrocytes due to the presence of the parasites. The 3D organization of a number of structures within the red blood cells, such as membrane profiles, possibly originating from the parasitophorous vacuole, and within the parasite itself, such as a polymorphic nature of hemoglobin-filled tubules and multi-lobular nuclei, was revealed. Taken together, the results show the contribution of FIB-SEM to the understanding of the 3D architecture of red cells and infectious agents as well as its potential as a complementary 3D electron microscopy tool to study the complexity of different biological models.

## Results

**Figure 1:**
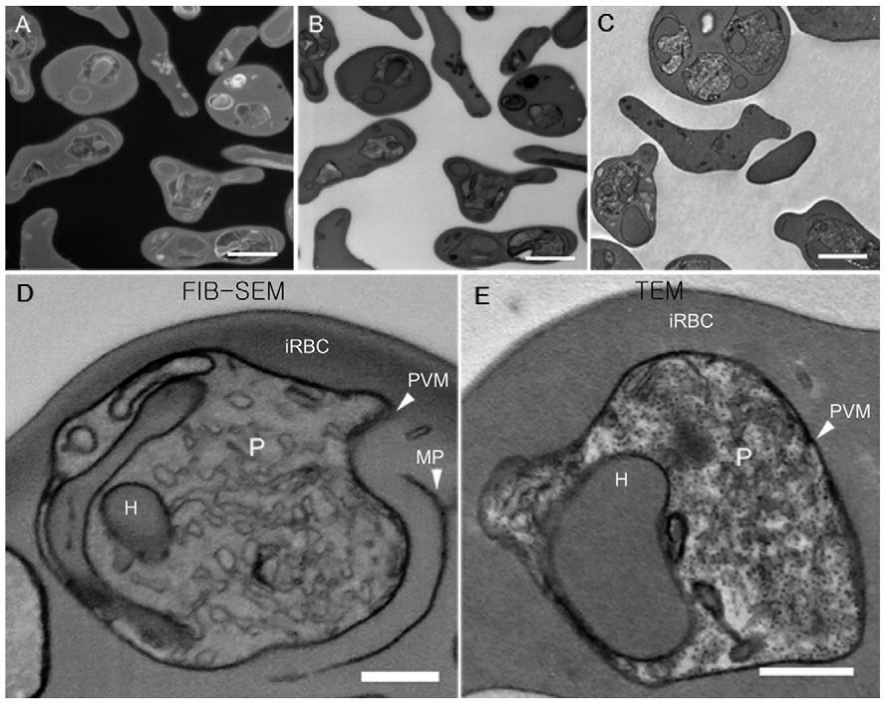
Comparison between images obtained from the surface of epoxide blocks containing red cells and transmission electron microscopy images of thin sections. (A) Image of an epoxide block surface containing infected erythrocytes obtained at 5 KeV detecting backscattered electrons. Note that membranes and other structures within the cells generate more signal (gray-white regions) in contrast to the dark background. Pixel size was 6.07 nm with an image size of 2048×1768 pixels. (B) Image shown in A after contrast inversion. Note that the contrast aspects of the image are similar to TEM images (C). (D) Backscattered image of a block surface at higher magnification. Infected erythrocytes were imaged at 2 KeV, the pixel size was 3.5 nm with an image size of 1024×943 pixels. (E) Image of an equivalent material obtained by TEM of a thin section. Bars represent 2 µm (A–C) and 500 nm (D–E). iRBC, infected red blood cell; P, parasite; PVM, parasitophorous vacuole membrane; H, hemoglobin filled compartment; MP, membrane profile.

The use of FIB-SEM to ion-mill and image the surface of a block containing infected red blood cells generated data from a large number of cells simultaneously (Figure 1 A–B). Osmium-stained membranes are generally easily visualized using a back-scattered electron detection system, where the presence of the metal increases the amount of signal detected. Images obtained presented a negative aspect when compared with TEM images (Figure 1A) which, once digitally inverted (Figure 1B), showed a similar aspect to the images obtained by TEM (Figure 1C). Observation of the same samples at higher magnification both by FIB-SEM (Figure 1D) and TEM (Figure 1E) showed similar information, although given the proper orientation of the sample, the visualization of the membrane bilayer was possible only in TEM images (not shown).

**Figure 2:**
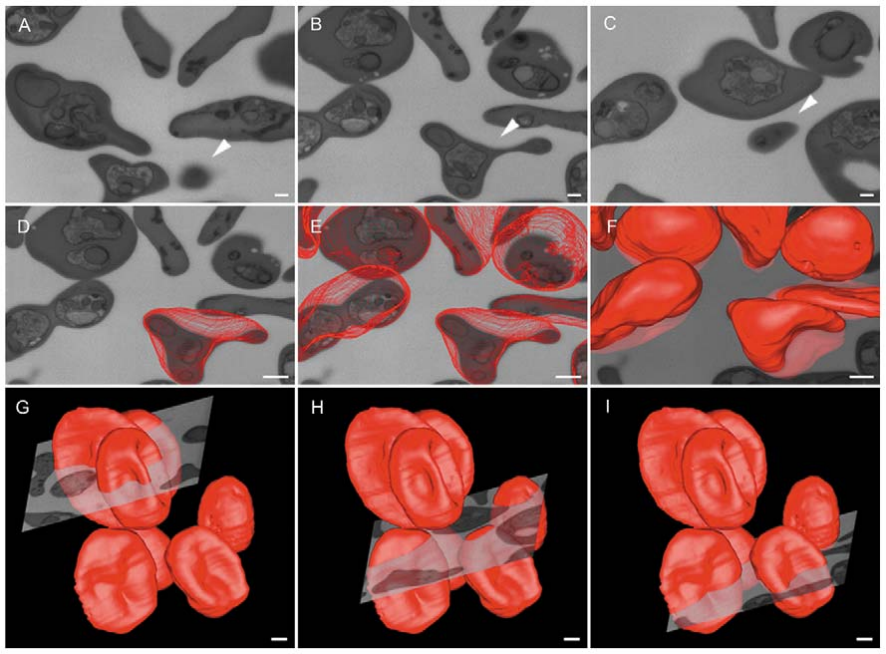
FIB-SEM reconstruction of multiple red cells simultaneously. (A–C) Images of the block face where the whole thickness of one cell was milled. (D–I) Reconstruction and modeling of multiple erythrocytes contained in the volume. Bars represent 500 nm (A–F) and 2 µm (G–I).

As the milling is made within the microscope chamber, it is possible to control the milling thickness and the number of slices so that the sampled volume obtained remains mainly limited by the quantity of material and the time available. Figure 2 shows sequential images from the block face (see Movie S1), where the whole thickness of one of the cells shown in Figure 1 was covered (Figure 2A–C, arrowheads). Since the images were made from the block face, it was possible to register many cells at the same time and, consequently, to reconstruct more cells from the image stack, either using contrast threshold (Movie S2) or manual segmentation (Movie S3) methods, as shown in Figure 2 (D–I). As several cells were reconstructed, and it was possible to obtain information on their 3D organization and to retrieve statistical data after analysis of their morphometric parameters such as surface area, volume and volumetric density (the volume occupied by specific structures within the cells) (Table 1).

**Table 1:** Morphometric analysis of the structures reconstructed from FIB-SEM images.

	Number per cell	Surface Area (nm^2^×10^6^)	Volume(nm^3^×10^8^)	Volume occupied in the erythrocyte (%)	Volumeoccupied in the parasite (%)
**Erythrocytes**	-	73±5	343±24	-	-
**Parasites**	2±1	17±1	48±5	14±2	-
**HCT**	1±0	9±1	9±1	-	19±1
**MS**	2±1	4±1	7±2	2±1	-

Values are expressed as means values ± standard error of the mean (SEM); Statistical significance was determined by Student's *t* test (P<0.05). Erythrocytes, n = 10; Parasites, n = 23; Hemoglobin-containing tubules (HCT), n = 23; Membranous structures (MS), n = 15.

**Figure 3:**
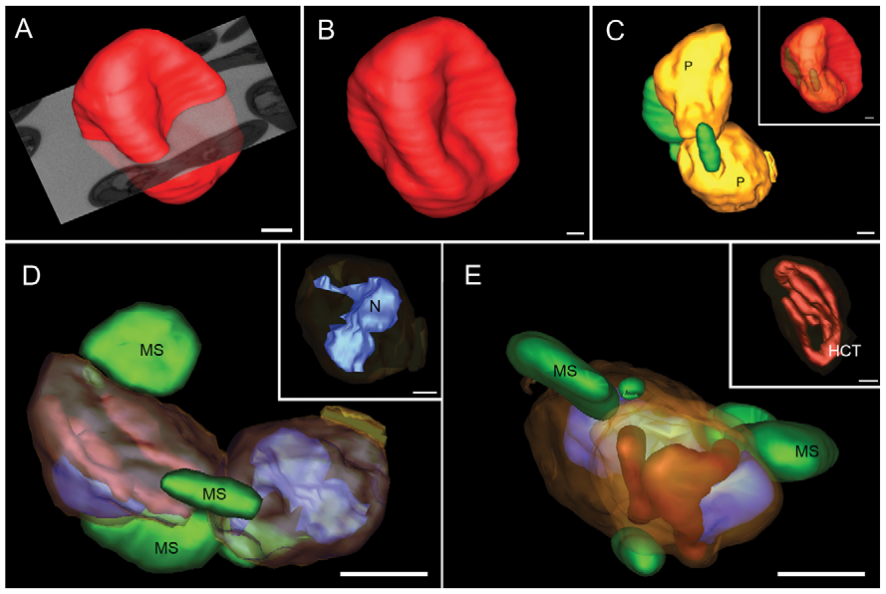
3D reconstruction by FIB-SEM shows surface morphology and internal details of an infected red cell. (A–B) 3D model of the surface of an infected erythrocyte showing deformations as a consequence of the presence of the parasite. (C) Modeling of parasites located underneath (PVM-yellow) and membranous structures (green). (C-inset) Semi-transparent iRBC, showing that the deformations on the RBC surface coincide with the presence of the parasites. (D, E) Different views showing the three-dimensional organization of the parasites inside a parasitophorous vacuole (PV, yellow), parasite membrane (purple, in D), membranous structures (MS, green), multi-lobular nuclei (D, inset, blue) and hemoglobin-containing tubules (HCT, E, inset, dark red). Bars: A, D and E, 1 µm. B–C, 500 nm.

Here, 180 sections with a thickness of 113.5 nm were cut and the block face was recorded in the BSE mode with a pixel size of 6.07 nm, resulting in a total volume of 2.7×10^3^ µm^3^ (∼20×12×10 µm). The reconstructed 3D architecture of *Plasmodium chabaudi* infected-erythrocytes was modeled and the resulting models showed deformations on the surface of the red cells due to the presence of the parasites (Figure 3A–C). Note that “swollen” regions on the surface of the iRBC coincide with the presence of the parasite (Figure 3B–C and C, inset). The three-dimensional organization of a number of structures, such as membranous structures in the cytoplasm of the infected erythrocyte (MS, green), a multi-lobular nuclei (N, Figure 3D, inset) and the polymorphic organization of an hemoglobin-containing tubule (HCT, Figure 3E, inset) were revealed (see Movie S4).

**Figure 4:**
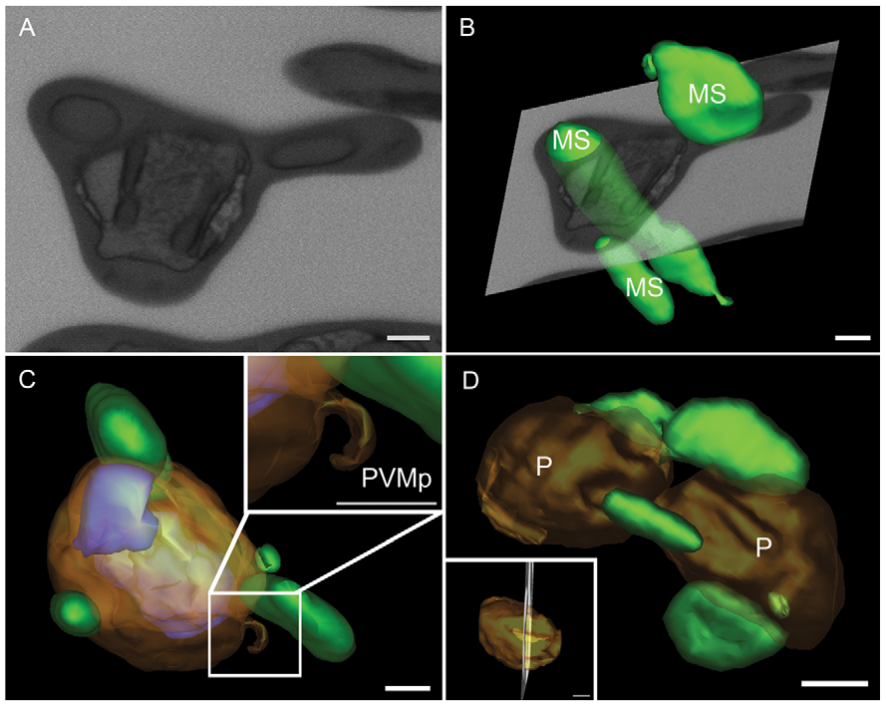
3D models of membranous structures and projections of the PVM within the red blood cell. (A) Membrane profiles delimiting internal spaces in the cytoplasm of an infected erythrocyte. (B) Modeling of each structure (MS, green) showing their three dimensional organization and heterogeneity in shape and size (B–D). (C) Model showing projections of the PVM that may contribute to the formation of a TVN (PVMp, inset). (D) Projections from the PVM visualized in cross section (approx. 10 cross section images) P, parasite. Bars represent 500 nm (A–C), 1 µm (D) and 500 nm (D, inset).

3D reconstruction of *P. chabaudi* infected cells revealed the presence of membranous structures enclosing the hemoglobin present in the cytoplasm of the erythrocyte and membrane projections from PVM, which may contribute to the formation of the TVN described in other *Plasmodium* species (Figure 4). Maurer's cleft-like structures were also present, as seen by electron tomography (not shown), but too small to be reconstructed in the FIB series obtained, given the slice thickness used. In a single image of the block surface (Figure 4A) or in its corresponding image obtained from thin sections in a TEM (not shown), these membranous structures appeared as rounded membranes with their interior filled by the erythrocyte cytoplasm (Figure 4A). Three-dimensional reconstruction showed that these structures were heterogeneous in shape and size (Figure 4B). In addition, projections from the PVM (Figure 4C, Figure S1) were also visualized, especially when cross section images of such structures were obtained (approx. 10 cross section images in this model) (Fig. 4D, inset). Membranous structures in this model occupied 2% of the volume of the erythrocyte (Figure 4D; Table 1).

**Figure 5:**
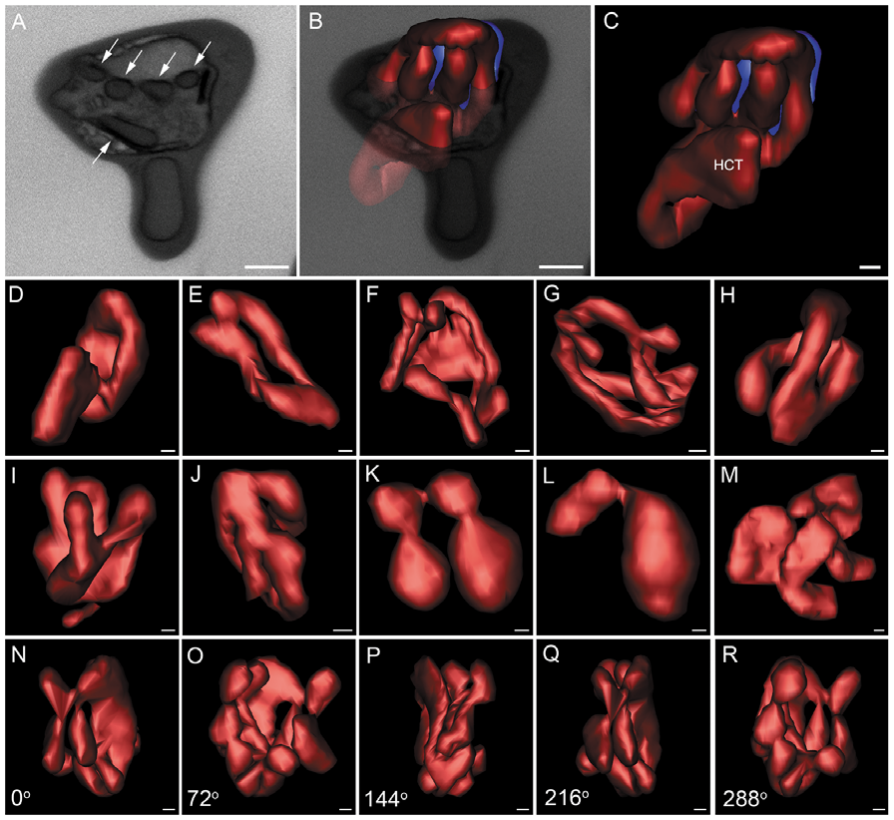
Modeling of multiple cells reveals a polymorphic aspect of hemoglobin-containing tubules in all parasites. (A) Vesicular aspect of endosomes in a single image of the block surface (arrows). (B, C) Interconnected tubular aspect of a parasite hemoglobin-containing compartment. (D–M) Polymorphic characteristics of the parasite hemoglobin-containing tubules (HCT) in different cells. Note that such structures appear as a single compartment in all cells analyzed. (N, R) Rotation of a single tubular compartment around the x axis. Bars represent 500 nm (A–C) and 200 nm (D–R).

Another feature revealed by 3D modeling of *Plasmodium chabaudi* infected-erythrocytes was the structure formed during internalization of portions of the erythrocyte cytoplasm, a mechanism that is believed to be important for the later formation of the food vacuole. Figure 5A shows profiles of hemoglobin-filled “vesicles” in a single image of the block surface. As in TEM, in such images they appear as cytoplasmic vesicles without an apparent connection. After reconstruction, connections between the vesicles were seen, where they appear as a unique fully interconnected tubular network (Figure 5 B–C). This was found in all reconstructed cells (Figure 5D–M). Rotated 3D models (360 degrees on the × axis) showed the complex organization of this structure at different stages of assembly/disassembly (Fig. 5 N–R). The average volume of these structures was around 9×10^8^ nm^3^, which represents approx. 19% of the volume of the parasite (Table 1).

**Figure 6:**
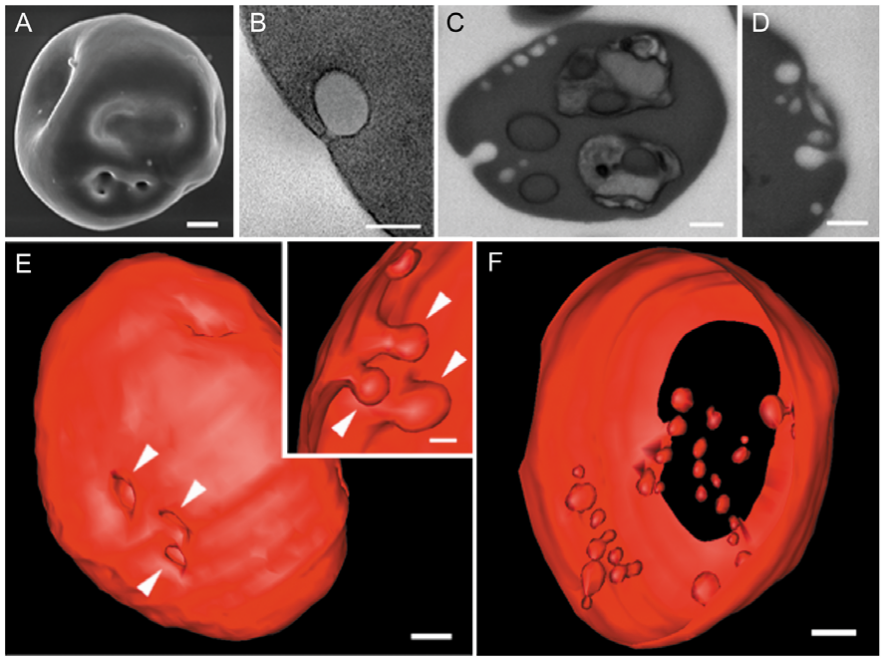
Simultaneous visualization of surface and intracellular structures by FIB-SEM. (A) Scanning electron micrograph of a *Plasmodium chabaudi* infected erythrocyte. Note the deformations in the erythrocyte membrane due to the presence of the parasite. (B) Transmission electron micrograph of a vesicle derived from the plasma membrane of the erythrocyte. (C, D) Similar vesicles were observed in the serial images obtained with FIB-SEM. (E) 3D model of an infected-erythrocyte showing similar deformations seen on the surface of the erythrocyte membrane by field emission scanning electron microscopy (A). (E-inset) Internal aspect of the membrane invaginations seen from the external perspective in E. (F) Visualization of inner portions of the erythrocyte showing vesicles of different sizes, presumably originated from membrane invaginations. Bars represent 500 nm (A), 200 nm (B), 500 nm (C), 500 nm (D), 500 nm (E), 500 nm (F) and 250 nm (E, inset).

Field-emission scanning electron microscopy analysis of infected erythrocytes also showed deformations in the erythrocyte membrane, where invaginations on the surface of the host cell could be clearly seen (Figure 6A). These structures were visualized by transmission electron microscopy (Figure 6B) and single images of the block surface (Figure 6C–D) as vesicles derived from the plasma membrane of the erythrocyte. As the cells present in the milled volume were randomly oriented, images obtained presented different section plans of the erythrocyte membrane (and other structures). In the sequence of images acquired they appeared in cross sections, being easily visualized, and in longitudinal sections, where their visualization is limited. Nevertheless, the overall aspect of the membrane invaginations and vesicles could be defined by a combination of information obtained in cross/longitudinal sections. In this regard, analysis of FIB-SEM sequences allowed the combination of surface analysis with the visualization of the sub cellular domain below these regions, which was shown to contain vesicles that have different shapes and sizes (Figure 6E–F) and suggested that these invaginations may lead to the formation of cytoplasmic vesicles. In the reconstructed models, the invagination of the erythrocyte membrane and the vesicles distributed along the cytoplasm of infected-erythrocyte could be seen in different subcellular domains of the red cell (Figure 6E–F).

## Discussion

Three-dimensional visualization of infected erythrocytes and *Plasmodium* is a key point for an accurate comprehension of the complex ultrastructural organization of the interactions between the parasite and the red blood cell. As the erythrocyte has no intracellular organelles or trafficking machinery, *Plasmodium* constructs an entirely novel trafficking system for protein and antigen transport. This secretory system has been extensively studied in *P. falciparum*, where the Maurer's clefts and a tubovesicular network were characterized three-dimensionally [38]. The formation of a tubovesicular network and clefts in a cistern shape resembling the Golgi apparatus was also described in erythrocytes infected with *P. vivax* [39] and *P. berghei* [7]. However, 3D organization of these structures has been studied only in *P. falciparum* [40]. Here, we show the three-dimensional organization of membranous structures and projections of the PVM. Given the similarity of our results with those that describe the tubovesicular network in other *Plasmodium* species [8], it is possible that these projections may correspond to a precursor of the *P. chabaudi* TVN. In addition, as the membranous structures (MS) found in the cytoplasm of the iRBCs enclose portions of hemoglobin, it is also possible that such compartments may originate from projections of the parasitophorous vacuole, although a connection between both structures has never been observed. It is also possible that such connections between the PVM, TVN and MS may occur in other moments of the differentiation process of the parasite during its life cycle.

The simultaneous observation of internal compartments, compartments that communicate with the plasma membrane, and the plasma membrane itself (cell surface), at high resolution, is of extreme importance in samples where the disease introduces modifications in the intracellular environment and extracellular face of the host cell (Figure 6E–F). Morphological changes in infected erythrocytes induced by the presence of *Plasmodium* species have been described and include the formation of invaginations and vesicles on the surface of the red blood cell, as well as the formation of micro and small cytoplasmic vesicles [41]. An association between these structures has been suggested. Sterling et al [42] described micro vesicles in the cytoplasm of erythrocytes infected with *Plasmodium simium*, and proposed that they were fusing with the plasma membrane of red blood cells, thereby increasing the surface of host cells [42]. The association between these cytoplasmic and surface structures, however, can be better visualized observing an infected red blood cell in three dimensional models.

**Figure 7:**
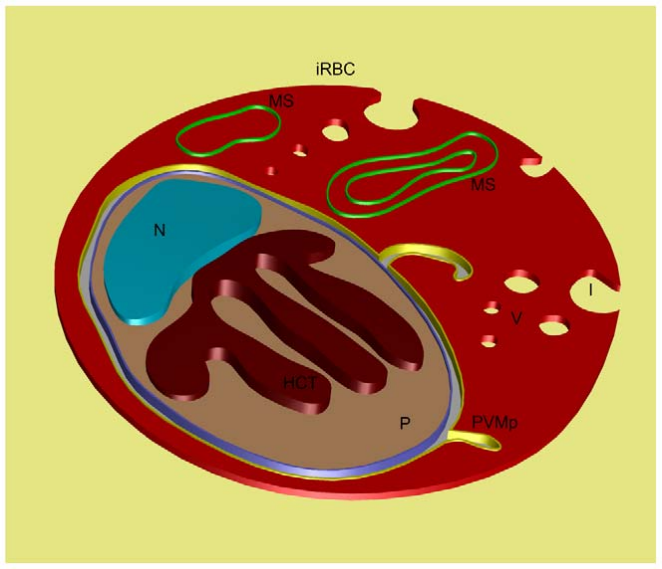
Schematic model summarizing the structure of *Plasmodium chabaudi* infected red blood cell. (iRBC) Infected red blood cell; (I) Invaginations of the plasma membrane of the iRBC; (V) Vesicles distributed along the cytoplasm of the iRBC; (MS) Membranous structures enclosing the hemoglobin; (PVMp) Projections of the PVM; (P) Parasite; (HCT) Hemoglobin-containing tubules (only closed tubules are presented); (N) Nucleus.

A schematic drawing that summarizes the data obtained is shown in Figure 7, where an infected red blood cell presents invaginations of the plasma membrane and vesicles distributed along the cytoplasm. Membranous structures enclosing the hemoglobin in the cytoplasm of the infected erythrocyte and projections of the PVM are shown. The parasite internalizes portions of the erythrocyte cytoplasm forming a network of interconnected tubules filled with hemoglobin. Closed tubules are represented, but open tubules were also found.

Surface analysis of a block face of epoxide-embedded osmium-stained materials using back scattered electrons (FIB-SEM) has been shown to provide information on the structural organization of a variety of models [35], from tissues [43], [34] to viruses [44]. Recently, FIB-SEM was applied to reconstruct nuclei of *P. falciparum* (rings, trophozoites and schizonts) showing different patterns of sub-nuclear organization [45]. Improved methods for three-dimensional visualization of biological samples are continuously being developed, giving important contribution to the understanding of the complex intracellular organization. Among the already large repertoire of 3D reconstruction techniques, recently reviewed for malaria biology [46], FIB-SEM appears as a novel technique that can retrieve high resolution 3D information of large volumes. Regarding the 3D organization of infected red blood cells, novel techniques such as cryo transmission X-ray microscopy were recently applied to describe a membrane network secreted by the parasite into the cytoplasm of the infected erythrocyte [40]. Cryo transmission X-ray microscopy, a method that exploits the natural contrast of biological samples, has been used to image intracellular malaria parasites with spatial resolution in the order of 50 nm [47], [48]. In addition, serial sectioning followed by electron tomography was used to generate whole cell images with a full depth of 6–8 µm corresponding to an RBC infected with *P. falciparum* (using electron tomograms of 27 serial sections). Another recent advanced technique used axial scanning transmission electron tomography to reconstruct four 1 µm serial sections, which allowed global 3D views of a whole parasite [23].

In this work, we showed the potential of FIB-SEM to reconstruct large volumes in a model of erythrocytes infected with malaria parasites (see Movie S5). The capacity of this technique to produce large volumes of information, suggest that FIB-SEM may be an alternative relevant approach to different studies involving the structural analysis of other biological models. The semi-automated image acquisition in FIB-SEM skips the intensive and time-consuming steps for serial-sectioning TEM due to operator involvement in cutting sections and gathering data. Furthermore, imaging of the block face also removes the need to deal manually with ribbons of fragile sections, and facilitates the alignment of the serial images, because there are no distortions or shrinkage.

Three-dimensional imaging of multiple cells in high resolution may also be important for statistical data, since morphometrical analysis based on the total volume of an elevated number of cells in the sample can be done. The 3D information obtained in this work from multiple red blood cells infected with *Plasmodium* generated statistical data on the 3D changes in erythrocyte architecture induced by the parasite, enabling the analysis of different morphometric parameters such as surface area, volume and diameter (Table 1).

Potentially, FIB-SEM can be combined with other techniques such as cryo preservation of the samples, enzyme cytochemistry, immunolabeling (pre-embedment labeling of surface exposed antigens for example) and the use of fluorescent markers followed by a photo-oxidation assay, providing insights into the 3D distribution of cellular components.

## Materials and Methods

### Ethics Statement

This study was approved by the Ethics Committee for Animal Experimentation of the Health Sciences Centre of the Federal University of Rio de Janeiro, under the Protocol n° IBCCF 129, according to the Brazilian federal law (11.794/2008, Decreto n° 6.899/2009) that is based on the “Guide for the Care and Use of Laboratory Animals” prepared by the National Academy of Sciences, USA, and the “Australian Code of Practice for Care and Use of Animal for Scientific Purpose”. All animals received humane care in compliance with the above mentioned guides used by the Ethics Committee to approve the protocol.

### Parasites

*Plasmodium chabaudi* (clone AJ, lethal) was maintained stored as stabilates in liquid nitrogen between experiments in order to avoid any selection of virulent strains. Parasitized mouse red blood cells (pRBC) from a liquid N_2_ preserved stabilate were injected into male mice (Swiss). After amplification, 1×10^6^ pRBC were inoculated intraperitoneally into individual mice to induce a regular experimental infection. Parasitemia, expressed as a percentage of infected erythrocytes, was monitored by Giemsa-stained tail-blood films, and determined in at least 500 red blood cells by microscopic examination. Mice with parasitemia higher than 80% were used.

### Transmission electron microscopy

pRBC were washed in PHEM buffer (2.5 mM MgCl_2_, 35 mM KCl, 5 mM EGTA, 10 mM Hepes, 30 mM Pipes), pH 7.2, fixed for 1 hour in a solution containing 2.5% glutaraldehyde, 4% formaldehyde, 4% sucrose in 0.1 M PHEM buffer, postfixed in 1% OsO_4_ plus 0.8% ferrocyanide and 5 mM CaCl_2_ in 0.1 M cacodylate buffer for 1 hour, dehydrated in ascending concentrations of ethanol and embedded in Polybed 812 epoxide resin. Thin-sections (70 nm) were obtained and stained for 40 min in 5% aqueous uranyl acetate, for 5 min in lead citrate and observed in a Jeol 1200EX electron microscope operating at 60 kV.

### Field-emission scanning electron microscopy

pRBC were washed in PHEM buffer (2.5 mM MgCl_2_, 35 mM KCl, 5 mM EGTA, 10 mM Hepes, 30 mM Pipes), pH 7.2, fixed for 1 hour in a solution containing 2.5% glutaraldehyde, 4% formaldehyde, 4% sucrose in 0.1 M PHEM buffer and settled onto poly-l-lysine-coated coverslips. Samples were than postfixed in 1% OsO_4_ plus 0.8% ferrocyanide and 5 mM CaCl_2_ in 0.1 M cacodylate buffer for 30 min, dehydrated in an ethanol series, and critical-point dried with CO_2_. Samples were ion-sputtered with a 2–3 nm carbon layer using a Gatan model 681 to avoid charge effect. Secondary electron images were obtained in a JEOL JSM 6340 F field emission scanning electron microscope at a 5.0 kV accelerating voltage.

### FIB-SEM

For observation in FIB-SEM, blocks of resin embedded samples were mounted on a support stub of the Nova 600 Nano Lab Dual Beam instrument (FEI Company). A 3 nm layer of platinum was deposited onto the sample, within the microscope chamber. Milling conditions were 30 kV acceleration voltage and a beam current of 1000 pA, in the SE imaging mode. The sample was tilted 52° to orientate the surface perpendicular to the Ga^+^ ion beam and a U-shaped trench was cut around the area of interest. For slice and view, the sample was tilted back to 0°, and the block was milled with the ion beam pointing at an angle of 38° to the sample surface, and the dwell time was 0.3 µs. After milling, the sample was ready for slice and view. The slice thickness varied from 49.2 nm to 113.5 nm. Images of the cell surface were taken with the electron beam at 1–5 kV acceleration voltage, beam current at 4000 pA and dwell time 6 µs, in the BSE imaging mode.

### Three-dimensional reconstruction and modeling

Models were constructed on computer running MIDAS and IMOD software (Boulder Laboratory, University of Colorado, Boulder, Colorado, USA) [49]. Image stacks were aligned using MIDAS. We used IMOD to stack the aligned images and the structures of interest were traced to provide a 3D representation. Using the IMOD mesh feature of IMOD, we joined the contours of each object to form a 3D model. Movies of these models rotating in space were made using Windows movie maker.

## Supporting Information

Movie S1 Serial images in Z. Note the large number of cells that appears within the sample volume.
(MOV)

Movie S2 Three-dimensional visualization of infected red cells segmented using threshold values that distinguish surface membranes and internal structures.
(MOV)

Movie S3 3D reconstruction of many different cells by manual segmentation from the image stack.
(MOV)

Movie S4 Simultaneous visualization of surface and internal features of infected red blood cells. The 3D animation first shows deformations on the surface of the erythrocytes and then a number of structures inside the infected cell, such as the membranous structures and the projections of the PVM, parasite profiles, hemoglobin-containing tubules and multi-lobular nuclei.
(MOV)

Movie S5 Complete movie showing a merge of the animations shown in supplemental materials 3, 4 and 5.
(WMV)

Figure S1 Parasitophorous vacuole membrane (A) and parasite membrane (B) are superimposed in almost all regions. The merge of these membranes (C) demonstrates the region where membranes are not superimposed (arrowhead), which corresponds to the projections of the PVM. It indicates that these projections are formed only by the PVM. Bars represent 500 nm.
(TIF)
