# Supplementary figures and images for: Visualizing the 3D Architecture of Multiple Erythrocytes Infected with Plasmodium at Nanoscale by Focused Ion Beam-Scanning Electron Microscopy
Source: PLoS One. 2012 Mar 14;7(3):e33445. doi: 10.1371/journal.pone.0033445 (PMC3303842; doi:10.1371/journal.pone.0033445)

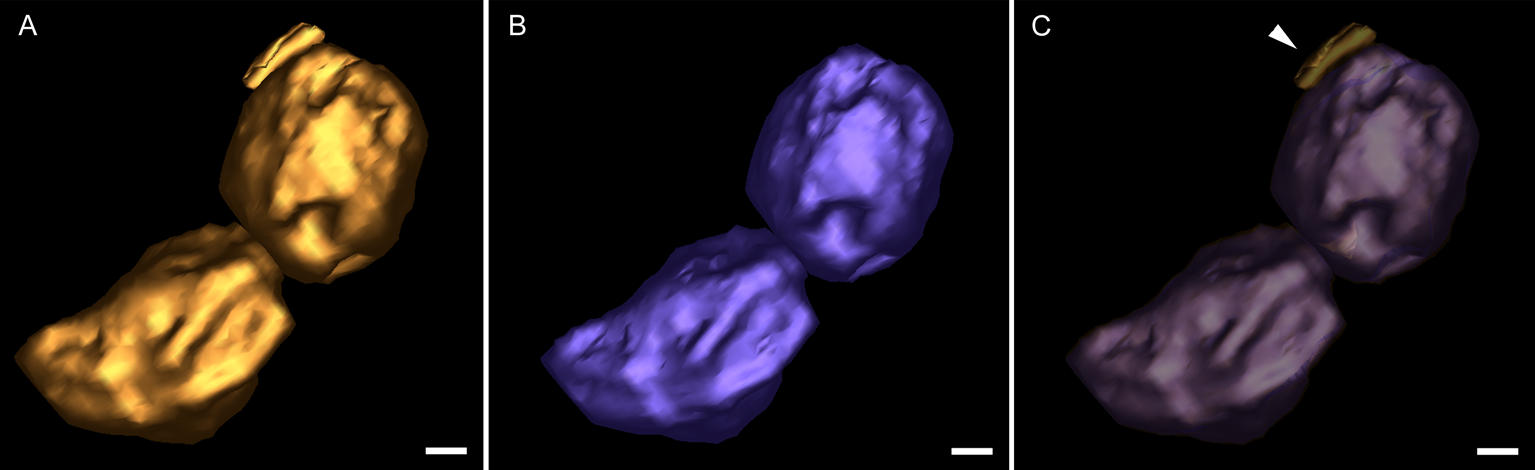

Supplement: Figure S1 — Parasitophorous vacuole membrane (A) and parasite membrane (B) are superimposed in almost all regions. The merge of these membranes (C) demonstrates the region where membranes are not superimposed (arrowhead), which corresponds to the projections of the PVM. It indicates that these projections are formed only by the PVM. Bars represent 500 nm. (TIF) [file pone.0033445.s006.tif]
